# Supplementary material for: A human respiratory tract-associated bacterium with an extremely small genome
Source: Commun Biol. 2021 May 26;4:628. doi: 10.1038/s42003-021-02162-6 (PMC8155191; doi:10.1038/s42003-021-02162-6)
Supplement: Supplementary file 2 — Supplementary information [file 42003_2021_2162_MOESM2_ESM.pdf]

Supplementary information

Title: A human respiratory tract-associated bacterium with an extremely small genome

Kazumasa Fukuda[1,*], Kei Yamasaki[2], Yoshitoshi Ogura[3], Toshinori Kawanami[2], Hiroaki Ikegami[2], Shingo Noguchi[2], Kentarou Akata[2], Keisuke Katsura[4], Kazuhiro Yatera[2], Hiroshi Mukae[5], Tetsuya Hayashi[6,*] & Hatsumi Taniguchi[1]

[1]Department of Microbiology, University of Occupational and Environmental Health, Japan, Kitakyushu, Fukuoka, Japan

[2]Department of Respiratory Medicine, University of Occupational and Environmental Health, Japan, Kitakyushu, Fukuoka, Japan

[3]Division of Microbiology, Department of Infectious Medicine, Kurume University School of Medicine, Fukuoka, Japan

[4]Frontier Science Research Center, University of Miyazaki, Miyazaki, Japan

[5]Department of Respiratory Medicine, Unit of Translational Medicine, Nagasaki University Graduate School of Biomedical Sciences, Nagasaki, Japan

[6]Department of Bacteriology, Faculty of Medical Sciences, Kyushu University, Fukuoka, Japan

*For correspondence; Kazumasa Fukuda and Tetsuya Hayashi.

Email: kfukuda@med.uoeh-u.ac.jp, thayash@bact.med.kyushu-u.ac.jp

Supplementary Figures (1–10)

Supplementary Tables (1–6)

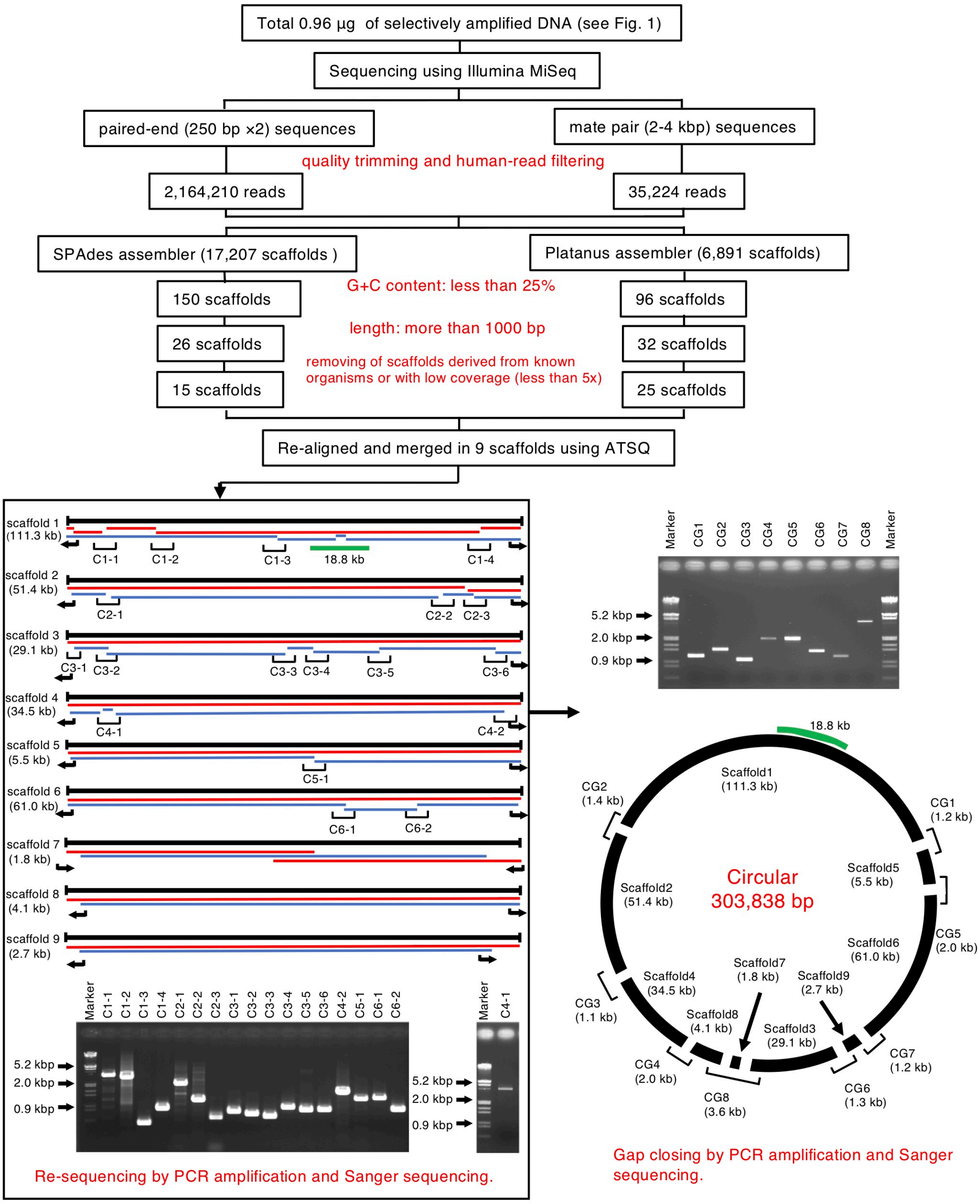

Supplementary Fig. 1. The process of IOLA genome re-construction. After filtering low-quality reads and human-derived read, Illumina reads were assembled using SPAdes and Platanus, respectively. Scaffolds longer than 1 kb and with GC contents less than 25% were selected. From the scaffold sets, those found to be derived from known organisms or with low coverage were removed, and the remaining scaffolds were used for constructing the final assembly. The green bar corresponds to the 18.8-kb region we previously determined (Ref. 9 in the main text). Red and blue bars represent the scaffolds generated by SPAdes and Platanus assemblers, respectively. Black bars represent the merged scaffolds using the ATSQ software. Locations of primers used for gap closing PCR are indicated by black arrows. Regions with any ambiguity in the merged scaffolds (C1-1 ~ C6-2) and gaps between the merged scaffolds (CG1 ~ CG8) were amplified by PCR, followed by 1.5% agarose gel electrophoresis analysis. Sequences of each PCR product were determined with Sanger sequencing.

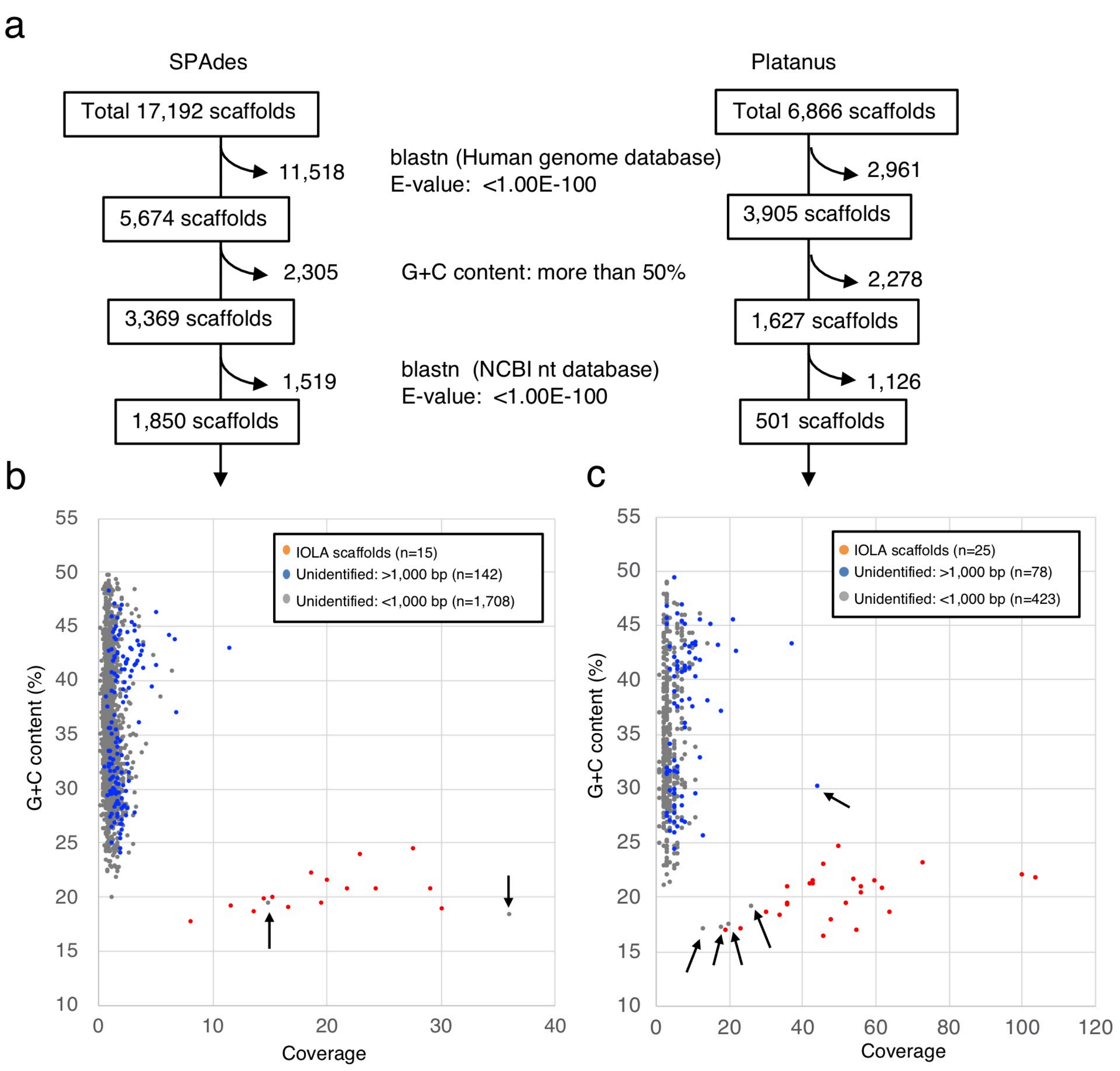

Supplementary Fig.2. Examination of the scaffolds which were not used in the final IOLA genome assembly. **a** We identified the scaffolds highly homologous to human sequences (GCF_000001405.39_GRCh38.p13) (threshold: E-value <1.00E-100), the scaffolds with a G+C content greater than 50%, and the scaffolds highly homologous to the sequences of known organisms (the latest non-redundant nucleotide sequence database; NCBI nt datadase) (E-value <1.00E-100) and excluded as scaffold not related to IOLA. **b** and **c** Sequence coverages and GC content of the remaining scaffolds were analyzed and compared to the scaffolds used in the final IOLA genome assembly. The seven scaffolds indicated by arrows were found to correspond to the gap regions observed during genome assembly (see Supplementary Fig. 1). These scaffolds were excluded from our genome assembly process due to the small sizes (<1 kb) or slightly higher GC content (>25%). Other scaffolds (most are smaller than 1 kb as indicated by gray dots) were clearly distinguished from the IOLA-derived scaffolds based on their sequence coverages and GC contents.

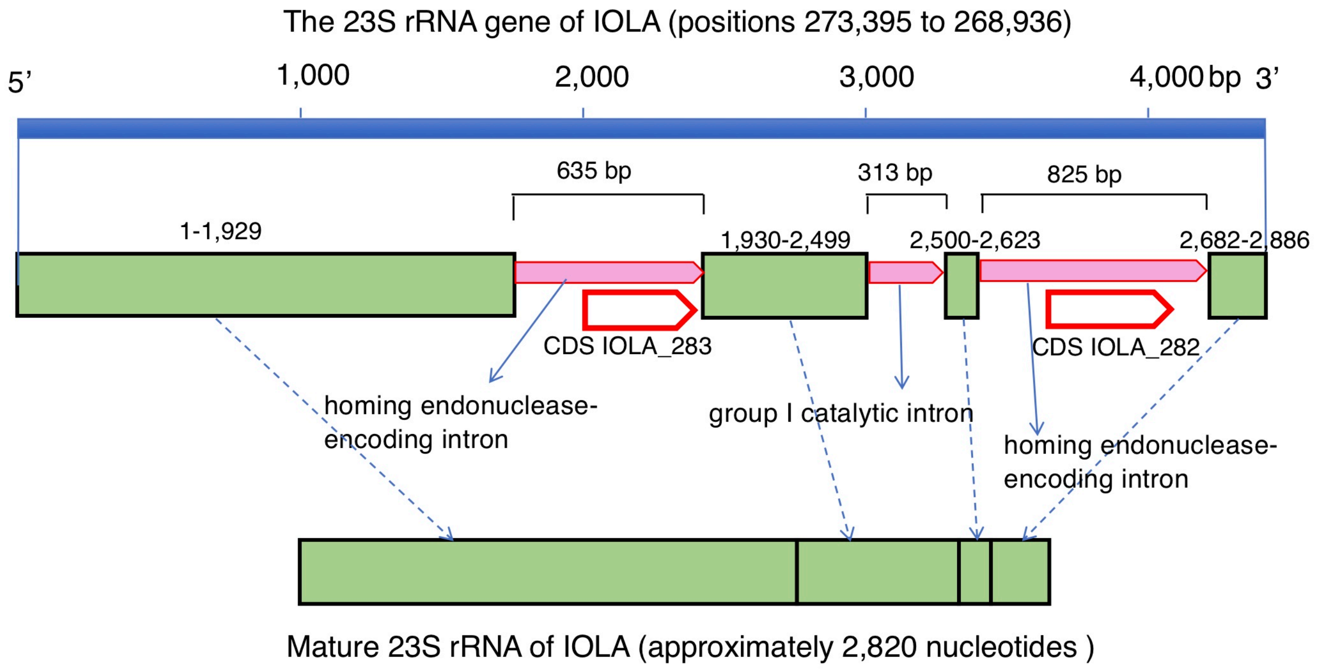

Supplementary Fig. 3. Schematic presentation of the 23S rRNA gene of IOLA. Green rectangles indicate the putative 23S rRNA sequence, and pink arrows indicate introns. The positions in the 23S rRNA sequence correspond to those in *Escherichia coli* K12. Two introns encoding homing endonucleases and a group I catalytic intron are shown. The secondary structures of IOLA rRNAs were analyzed using "Rfam 13.0" on the EMBL-EBI website (https://www.ebi.ac.uk/services/dna-rna).

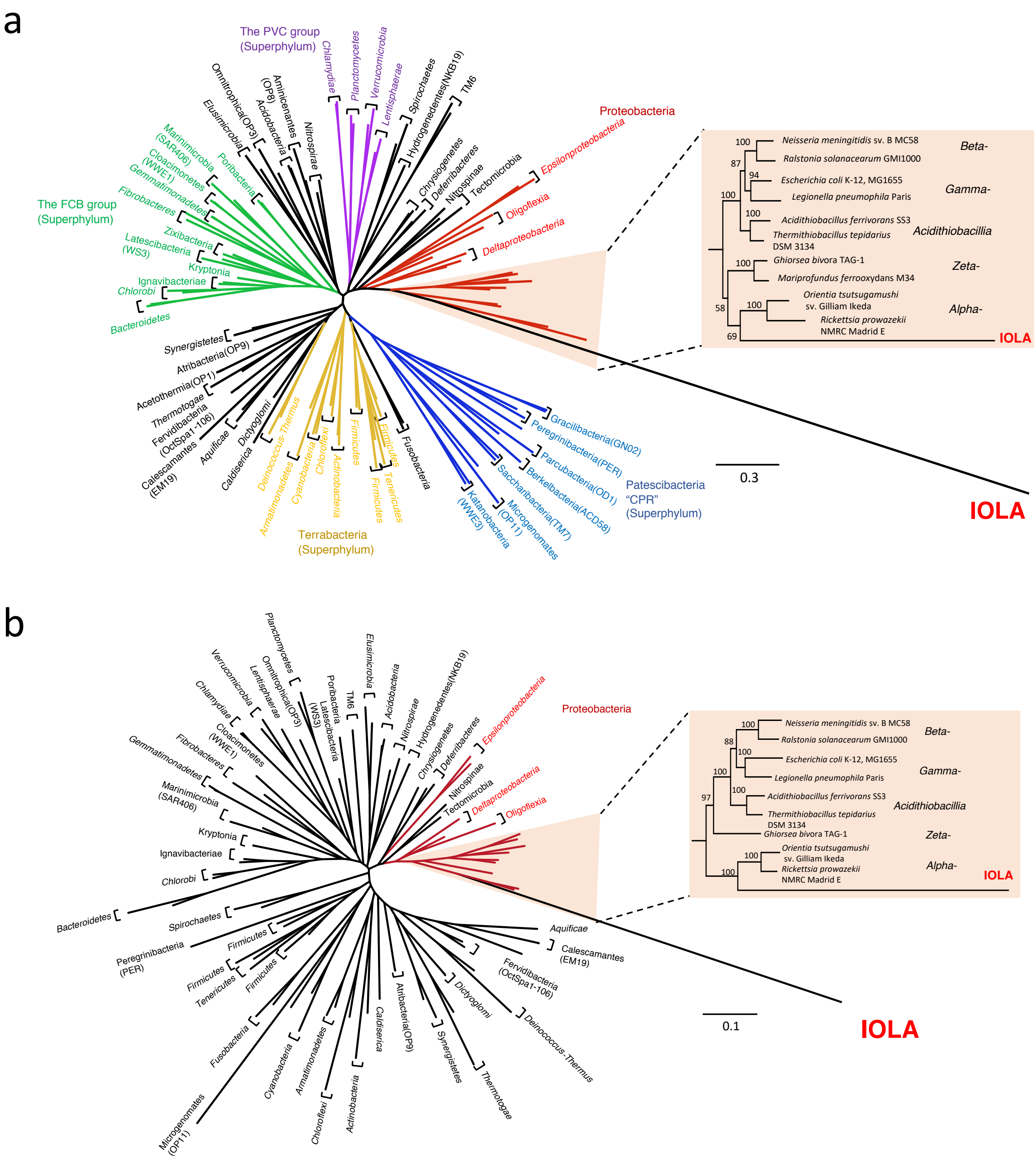

Supplementary Fig. 4. Maximum likelihood phylogenetic trees based on 16 ribosomal proteins and the 16S and 23S ribosomal RNA genes. a The tree based on the concatenated sequences of 16 ribosomal proteins. The tree includes 122 bacterial species (56 named bacterial phyla) and IOLA. A total of 123 sets of the 16 ribosomal proteins (RpL2, 3, 4, 5, 6, 14, 15, 16, 18, 22, and 24 and RpS3, 8, 10, 17, and 19) were used (listed in Supplementary Table 6). Candidate phyla are presented with nonitalicized names. Bacterial phyla belonging to the same superphylum are presented in the same color. The ML tree was constructed using the LG+R10 model and the 1,895-position alignment. **b** The tree based on the concatenated sequences of 16S and 23S rRNA genes. The tree includes 95 species that were selected from the 123 bacterial species analyzed in the panel based on the criterion that nearly full-length 16S and 23S rRNA genes were annotated (>1,200 bp and >2,000 bp, respectively). The ML tree was constructed using the TIMe+R10 model and the 3,421-position alignment. The scale bars indicate substitutions per site. Subtrees with bootstrap values (1,000 ultrafast bootstrap replicates) are shown in boxes.

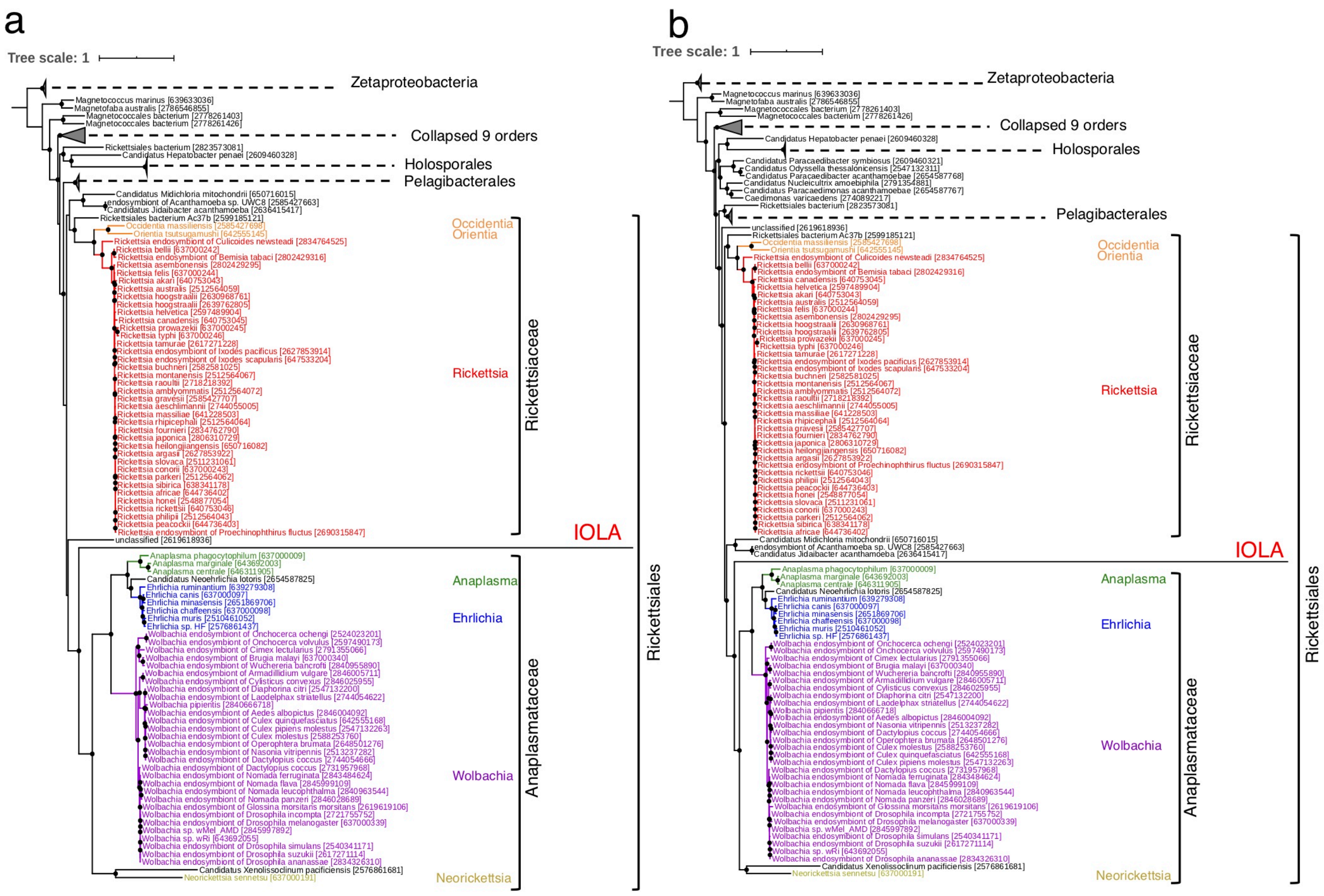

Supplementary Fig. 5. Maximum likelihood phylogenetic trees based on the concatenated sequences of ribosomal and other housekeeping proteins of class Alphaproteobacteria: Reconstruction of the trees shown in Fig. 4 using BMGE and PMSF. The ML trees shown in Fig. 4 were reconstructed using the BMGE and PMSF methods. The 2,121-position alignment of the 16 ribosomal proteins (a) and the 5,624-position alignment of 12 housekeeping proteins (b) prepared by BMGE were applied to construct ML trees using the PMSF model (LG+C20+F+R10) with 100 bootstrap replicates. Black dots indicate divergence episodes with bootstrap values greater than 80%. The scale bar indicates substitutions per site. In both trees, different genera belonging to order Rickettsiales are presented in different colors.

a

b

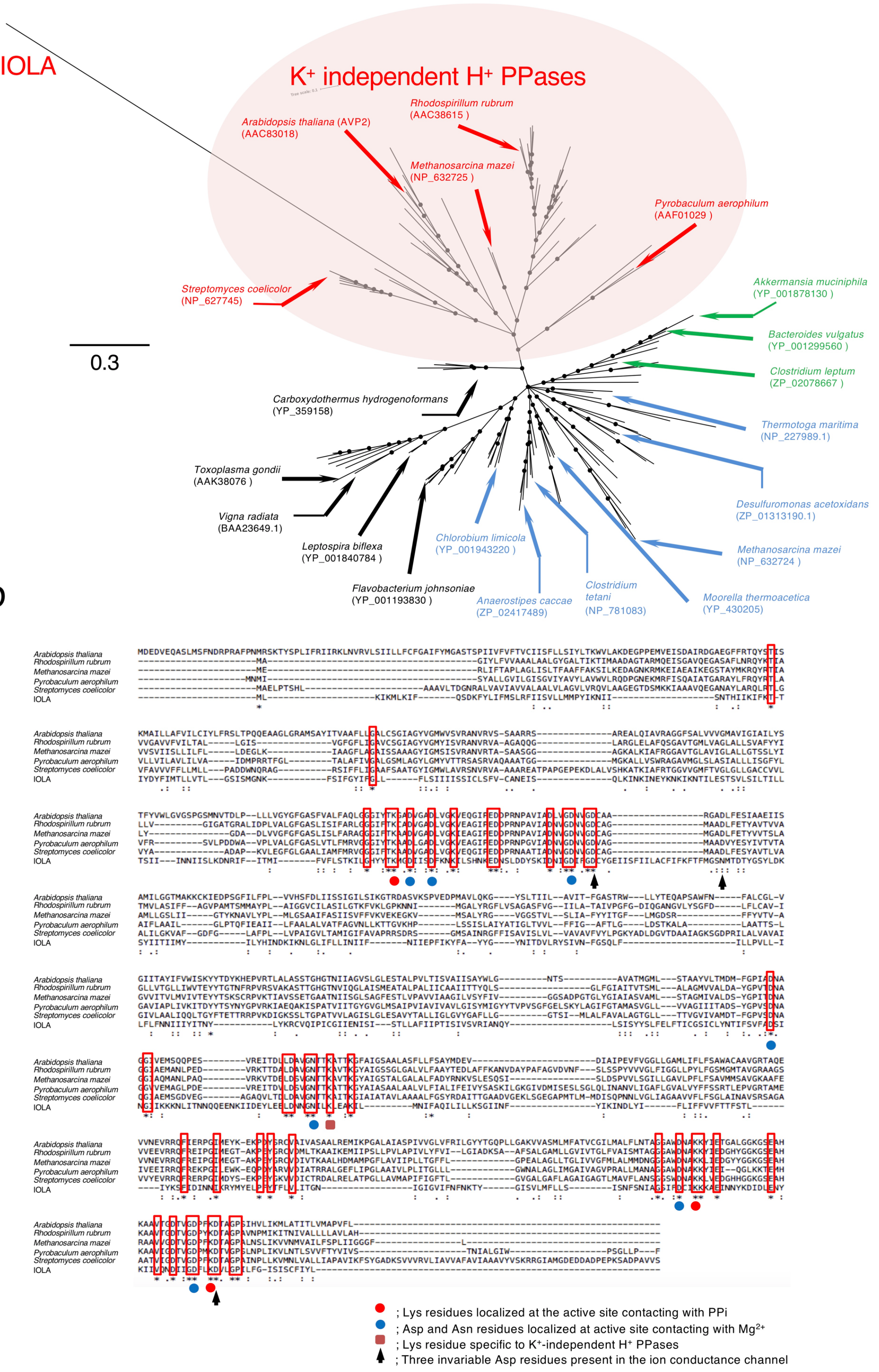

Supplementary Fig. 6. Phylogenetic analysis of the membrane PPase protein family. a The 121 PPase sequences used in a previous study[21] were obtained from the NCBI database and aligned with the PPase of IOLA. The alignment is composed of 345 residue columns. The phylogenetic tree was generated using MrBayes 3.2.6. Experimentally characterized K^+-independent H^+-PPases are indicated in red, K^+-dependent H^+-PPases

in black, Na^+-PPases in blue, and Na^+, H^+-PPases in green. The K^+-independent H^+-PPase cluster is indicated by light orange shading. Black dots indicate divergence episodes with bootstrap values greater than 80%. **b** Multiple-sequence alignment of K^+-independent H^+-PPases is shown. The sequences of K^+-independent H^+-PPases of *Arabidopsis thaliana* (accession no. AAC83018.1), *Rhodospirillum rubrum* (AAC38615.2), *Methanosarcina mazei* (NP_632725.1), *Pyrobaculum aerophilum* (AAF01029.1), *Streptomyces coelicolor* (NP_627745.1), and IOLA (in this study) were aligned using MAFFT. The residues that are completely conserved are highlighted by red boxes. The key residues for the function of this protein family are indicated by colored spheres. Three Lys residues in contact with PPi (red spheres) and six Asp residues and one Asn residue in contact with Mg^{2+} ions (blue spheres) are conserved in prokaryotic PPases. The Lys residue specific to the K^+-independent H^+-PPases is indicated by an orange square. Black arrows indicate three Asp residues invariably present in the ion conductance channel.

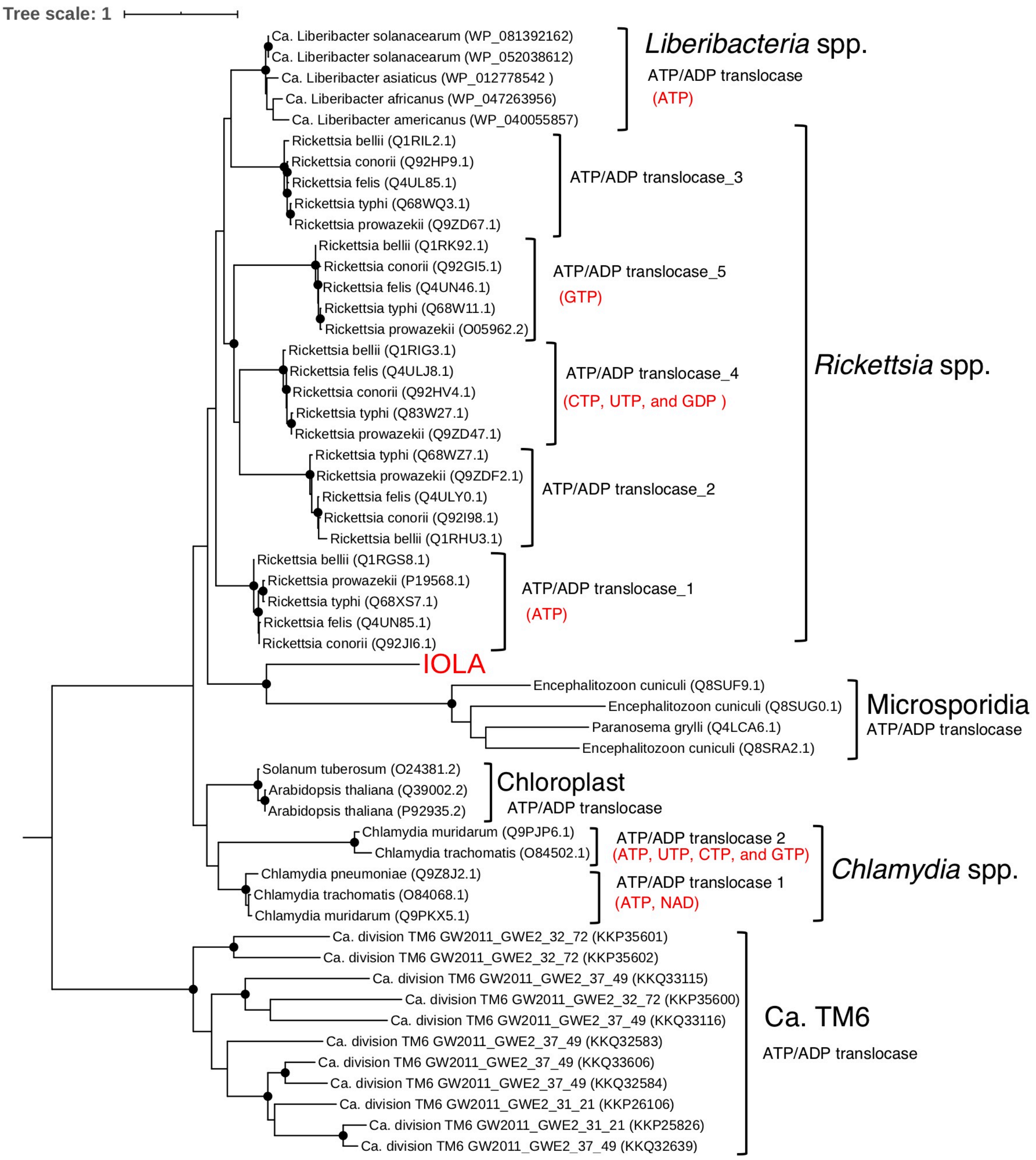

Supplementary Fig. 7. Phylogenetic analysis of the ATP/ADP translocase protein family. A total of 54 ATP/ADP translocase protein sequences, including 37 sequences identified by a blastp search of the UniProtKB/SwissProt database using the ATP/ADP translocase of IOLA as a query, 11 sequences of phylum TM6, and 5 sequences of *Ca.* Liberibacteria, were aligned with the sequence of the IOLA protein. The MAFFT alignment was trimmed by Gblocks 0.91b, and the resulting alignment (212 residue columns) was applied to construct an ML tree with the mtZOA+G4 model and 1,000 ultrafast bootstrap replicates. Black dots indicate divergence episodes with bootstrap values greater than 80%. The nucleotides that were experimentally confirmed to be translocated are indicated in parentheses in red.

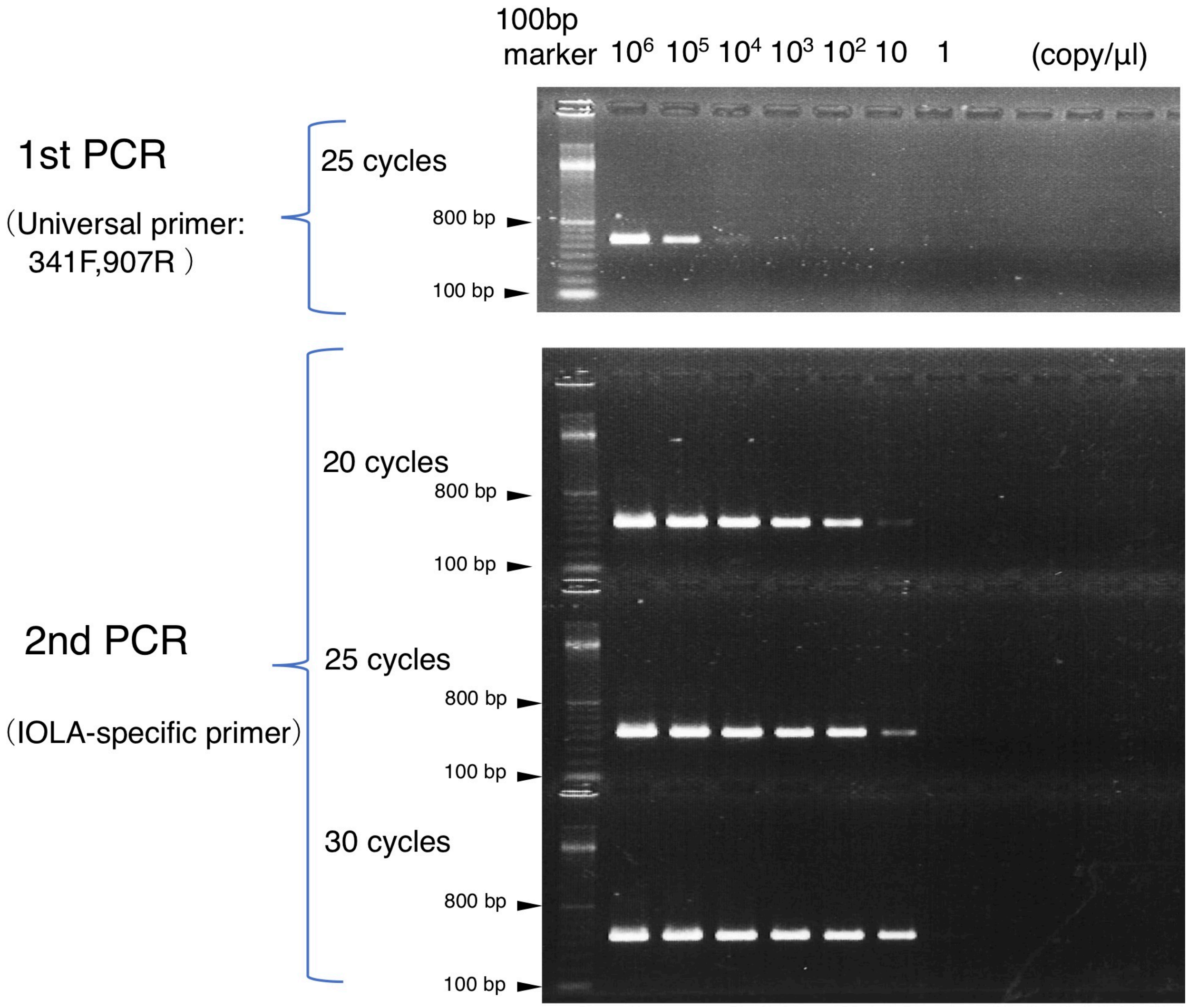

Supplementary Fig. 8. The detection limit of the IOLA-specific nested PCR targeting the 16S rRNA gene. The full-length 16S rRNA gene of IOLA was cloned into the pCR4-TOPO plasmid. Serially diluted plasmid DNA samples were subjected to nested PCR. PCR products were examined by 2% agarose gel electrophoresis. IOLA 16S rRNA-specific primers were described in our previous paper[9].

a

Amplicons	Position	SNPs											CDS	transsition/ transversion	S/NS
		KY282	KY923	KY90	KY519	KY440	KY798	KY494	CL22	KY207	KY677	KY405	(product)		(AA substitution)
PCR50	282	C	T	T	T	T	T	T	T	T	T	T	IOLA_047 (acriflavine resistance protein B)	Transition	NS (M toV)
	313	T	C	C	C	C	C	C	C	C	C	C	IOLA_047 (acriflavine resistance protein B)	Transition	S
	316	A	T	T	T	T	T	T	T	T	T	T	IOLA_047 (acriflavine resistance protein B)	Transversion	S
	318	G	A	A	A	A	A	A	A	A	A	A	IOLA_047 (acriflavine resistance protein B)	Transition	S
	331	T	A	A	A	A	A	A	A	A	A	A	IOLA_047 (acriflavine resistance protein B)	Transversion	S
	369	G	A	A	A	A	A	A	A	A	A	A	IOLA_047 (acriflavine resistance protein B)	Transition	S
	381	G	A	A	A	A	A	A	A	A	A	A	IOLA_047 (acriflavine resistance protein B)	Transition	S
	398	C	T	T	T	T	T	T	T	T	T	T	IOLA_047 (acriflavine resistance protein B)	Transition	NS (D to G)
	454	G	A	A	A	A	A	A	A	A	A	A	IOLA_047 (acriflavine resistance protein B)	Transition	S
	475	T	C	C	C	C	C	C	C	C	C	C	IOLA_047 (acriflavine resistance protein B)	Transition	S
	487	A	G	G	G	G	G	G	G	G	G	G	IOLA_047 (acriflavine resistance protein B)	Transition	S
	495	T	G	G	G	G	G	G	G	G	G	G	IOLA_047 (acriflavine resistance protein B)	Transversion	NS (H to N)
PCR100	711	T	T	T	C	C	T	T	T	T	T	T	IOLA_097 (DNA polymerase III subunit epsilon)	Transition	S
	970	T	T	T	C	C	C	C	T	C	C	T	IOLA_098 (hypothetical protein)	Transition	S
PCR125	216	T	T	T	T	G	T	T	T	T	T	T	IOLA_119 (hypothetical protein)	Transversion	S
PCR150	61	C	C	C	C	C	C	T	C	C	T	C	IOLA_156 (hypothetical protein)	Transition	S
	350	A	A	G	A	A	A	A	A	A	A	A	IOLA_156 (hypothetical protein)	Transition	NS (K to E)
	870	G	G	G	G	G	G	A	A	A	A	G	IOLA_157 (hypothetical protein)	Transition	NS (R to K)
PCR175	201	C	C	C	C	C	C	C	C	C	T	C	IOLA_188 (elongation factor Tu)	Transition	NS (D to N)
	358	C	C	C	C	C	C	C	C	T	T	C	IOLA_188 (elongation factor Tu)	Transition	S
	721	T	T	T	T	T	T	T	C	C	C	T	IOLA_188 (elongation factor Tu)	Transition	S
	768	T	C	T	T	T	T	T	T	T	T	T	IOLA_188 (elongation factor Tu)	Transition	NS (T to A)
	770	T	T	T	T	T	T	T	C	C	C	T	IOLA_188 (elongation factor Tu)	Transition	NS (N to S)
	810	T	T	T	T	T	T	T	T	T	G	T	IOLA_188 (elongation factor Tu)	Transversion	NS (N to H)
	939	C	C	C	C	C	C	A	C	C	C	C	IOLA_188 (elongation factor Tu)	Transversion	NS (A to S)
PCR250	27	T	T	C	T	T	T	T	T	T	C	T	IOLA_266 (hypothetical protein)	Transition	NS (N to S)
	28	T	T	T	T	C	T	T	T	T	T	T	IOLA_266 (hypothetical protein)	Transition	NS (N to D)
	30	G	G	G	G	A	G	G	G	G	G	G	IOLA_266 (hypothetical protein)	Transition	NS (T to I)
	31	C	C	C	C	C	C	C	C	C	C	T	IOLA_266 (hypothetical protein)	Transition	NS (T to A)
	47	C	C	C	C	C	C	C	C	C	A	C	IOLA_266 (hypothetical protein)	Transversion	NS (Q to R)
	48	T	T	T	T	T	T	T	T	T	C	T	IOLA_266 (hypothetical protein)	Transition	NS (Q to R)
	55	C	C	C	T	C	C	C	C	C	T	C	IOLA_266 (hypothetical protein)	Transition	NS (E to K)
	130	A	A	A	A	A	A	G	A	A	A	A	IOLA_266 (hypothetical protein)	Transition	NS (C to R)
	353	A	A	A	A	A	T	A	A	A	A	A	IOLA_266 (hypothetical protein)	Transversion	NS (N to K)
	517	T	T	T	T	T	T	C	T	T	T	T	IOLA_266 (hypothetical protein)	Transition	NS (S to G)

b

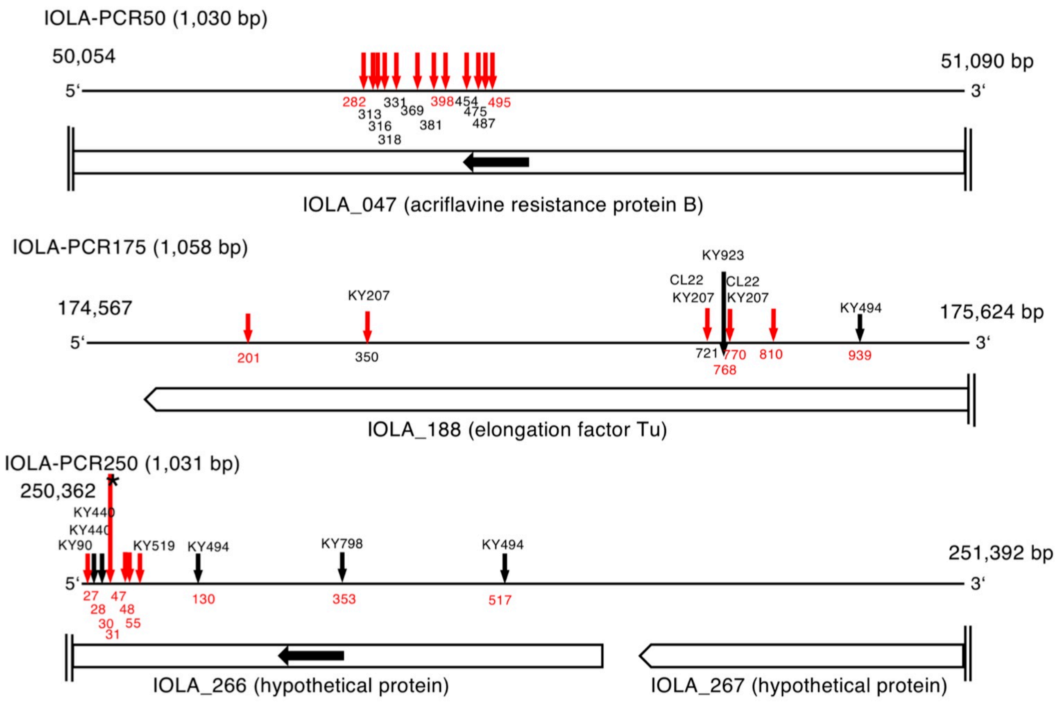

Supplementary Fig. 9. SNPs detected in the 12 genomic segments of IOLA obtained from 14 specimens. **a** Detailed information on all SNPs identified is shown. KY405 (identical to KY41, KY315, and KY366) was used as a reference. **b** The gene maps of segments PCR50, PCR175, and PCR250 and positions of SNPs identified in these segments are shown. Signs of recombination (accumulation of SNPs) were detected in these segments. CDSs are indicated by truncated boxes or arrows. Red vertical arrows indicate SNPs detected in segment PCR50 of KY282 and in segments PCR175 and PCR250 of KY677. When these SNPs were detected in other

IOLA samples, the sample names are indicated. Black vertical arrows indicate SNP sites that were detected in other IOLA samples (the sample names are indicated) but not in KY282 (segment PCR50) and KY677 (segments PCR175 and PCR250). The asterisk indicates the SNP that was detected in all specimens, excluding four samples obtained from a single patient (KY41, KY315, KY366, and KY405). SNPs that introduce nonsynonymous mutations are indicated by red position numbers.

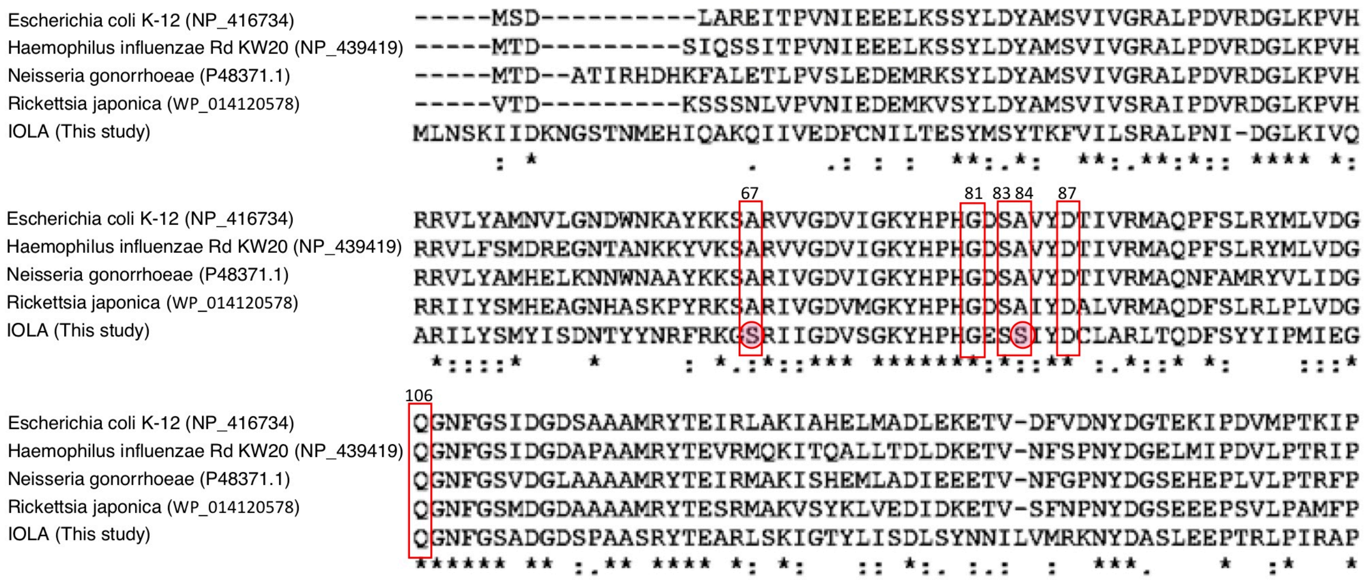

Supplementary Fig. 10. Multiple alignment of the amino acid sequences of GyrA. The amino acid sequences of GyrA from four representative fluoroquinolone-susceptible bacteria in phylum Proteobacteria, namely, *Escherichia coli* (gammaproteobacteria), *Haemophilus influenzae* (gamma), *Neisseria gonorrhoeae* (beta), and *Rickettsia japonica* (alpha), were aligned with that of IOLA (alpha) using MAFFT. The numbers in parentheses are the accession numbers of each sequence. The alignment of N-terminal sequences (corresponding to the 1-164 amino acid positions of *E. coli* K-12), which includes the quinolone resistance-determining region (QRDR), is shown. Six amino acid residues whose mutations are known to confer quinolone resistance in *E. coli* are indicated by red boxes. The numbers on columns correspond to the positions of the *E. coli* K-12 GyrA. Two IOLA-specific amino acid substitutions found in these residues are indicated by pink circles.

Supplementary Table 1. Comparison of IOLA with completely sequenced bacteria with extremely small genomes.

Bacterial strain	Phylum (Class)	Life style	Host	size (kb)	GC (%)	CDSs	Accession no.
Ca. Hodgkinia cicadicola TETCHI4	Proteobacteria (α)	Endosymbiosis	Insect (Cicada)	106	45.6	56	CP025310
Ca. Nasuia deltocephalinicola PUNC	Proteobacteria (β)	Endosymbiosis	Insect (Leafhopper)	112	16.6	138	CP013211
Ca. Vidania fulgoroideae OLIH	Proteobacteria (β)	Endosymbiosis	Insect (Leafhopper)	136	18.2	154	CP028360
Ca. Tremblaya phenacola TPMHIR1	Proteobacteria (β)	Endosymbiosis	Insect (Planococcus)	138	61.8	136	LN999011
Ca. Sulcia muelleri strain OLIH	Bacteroidetes	Endosymbiosis	Insect (Leafhopper)	157	24.9	185	CP010105
Ca. Carsonella ruddii HT	Proteobacteria (γ)	Endosymbiosis	Insect (Psyllid)	158	14.6	177	CP003544
endosymbiont of Rhynchophorus ferrugineus	Proteobacteria (γ)	Endosymbiosis	Insect (Rhynchophorus)	200	15.3	199	AP018161
Ca. Zinderia insecticola CARI	Proteobacteria (β)	Endosymbiosis	Insect (Clastoptera)	209	13.5	202	CP002161
endosymbiont of Euscepes postfasciatus	Proteobacteria (γ)	Endosymbiosis	Insect (Euscepes)	220	16.2	213	AP018159
endosymbiont of Pachyrhynchus infernalis	Proteobacteria (γ)	Endosymbiosis	Insect (Pachyrhynchus)	226	17.4	212	AP018160
endosymbiont of Sipalinus gigas	Proteobacteria (γ)	Endosymbiosis	Insect (Sipalinus)	233	17.7	233	AP018162
Ca. Uzinura diaspidicola str. ASNER	Bacteroidetes	Endosymbiosis	Insect (Scale insects)	263	30.2	227	CP003263
Ca. Portiera aleyrodidarum TV	Proteobacteria (γ)	Endosymbiosis	Insect (Whitefly)	281	24.7	269	CP004358
endosymbiont of Trabutina mannipara	Proteobacteria (γ)	Endosymbiosis	Insect (Whitefly)	298	32.6	246	LT594522
IOLA KY-405	Unclassified	Unknown	Unknown (Human?)	304	20.7	310	This study

Completely sequenced bacterial genomes were sorted by size using “Browse microbial genomes” on the NCBI website (https://www.ncbi.nim.nih.gov). Genomes smaller than 304 kb were selected. When multiple genomes had been sequenced in the same species, the strain first sequenced was chosen.

Supplementary Table 2. The results of secondary structure analysis of the 16S and 23S rRNA of IOLA using Rfam.

rRNA	ID	start/end	bits score	E-value	accession No.
16S rRNA gene (1,504 bases)	SSU_rRNA_bacteria	1/1504	1117.0	0	RF00177
	SSU_rRNA_archaea	5/1502	772.8	3.50E-228	RF01959
	SSU_rRNA_microsporidia	5/1499	608.6	1.00E-184	RF02542
	SSU_rRNA_eukarya	5/1499	573.5	1.10E-171	RF01960
23S rRNA gene (4,460 bases)	LSU_rRNA_bacteria	3/3450	1572.5	0	RF02541
	LSU_rRNA_archaea	2/3466	1030.9	0	RF02540
	LSU_rRNA_eukarya	150/3668	682.9	1.60E-172	RF02543
	Intron_gpI	2987/3247	64.1	1.30E-14	RF00028

The secondary structures of IOLA rRNA were analyzed using “Rfam 13.0” on the EMBL-EBI website (https://www.ebi.ac.uk/services/dna-rna).

Supplementary Table 3. The results of homology searches of the IOLA 16S rRNA gene.

Database	Description	Identity	coverage	E-value	Accession No.
Nucleotide collection (nr/nt)	Uncultured bacterium clone Nit2Au0637_374	73%	92%	0	FJ628217.1
	Uncultured bacterium clone SIP13C_6C	73%	84%	3.00E-177	KF741419.1
	Uncultured proteobacterium clone SCM117	71%	91%	7.00E-173	KX363698.1
	Uncultured alpha proteobacterium clone SCM111	71%	91%	7.00E-173	KX363697.1
	Uncultured bacterium clone: Ms-32-St1w-2-091	73%	78%	2.00E-172	AB990226.1
16S rRNA_typestrains	Bartonella australis strain Aust/NH1	71%	84%	3.00E-158	NR_102494.1
	Liberibacter crescens strain BT-1	71%	84%	1.00E-157	NR_102476.2
	Bartonella australis strain Aust/NH1	71%	77%	1.00E-157	NR_115816.1
	Ehrlichia ewingii strain Stillwater	72%	77%	1.00E-157	NR_044747.1
	Bartonella rattaustraliani strain AUST/NH4	71%	77%	5.00E-156	NR_116175.1
Refseq representative genomes	Wolbachia endosymbiont strain TRS	70%	91%	6.00E-157	NC_006833.1
	Bartonella australis Aust/NH1	71%	84%	3.00E-155	NC_020300.1
	Liberibacter crescens BT-1	71%	84%	9.00E-155	NC_019907.1
	Bartonella rochalimae ATCC BAA-1498 supercont1.2	71%	84%	1.00E-153	NZ_KL407338.1
	Bartonella rochalimae ATCC BAA-1498 supercont1.3	71%	84%	1.00E-153	NZ_KL407339.1
HTGS	Uncultured SAR11 cluster alpha proteobacterium H17925_38M03	71%	77%	7.00E-151	GU574703.1
	Uncultured SAR11 cluster alpha proteobacterium H17925_48B19	70%	92%	2.00E-145	GU574705.1
	Uncultured Rhodospirillales bacterium HF0500_02H05 clone HF0500_02H05	70%	82%	4.00E-141	GU567985.1
	Uncultured SAR11 cluster alpha proteobacterium H17925_23J24	70%	77%	6.00E-139	GU574702.1
	Uncultured nuHF1 cluster bacterium HF0770_35I22 clone HF0770_35I22	70%	82%	7.00E-132	GU568015.1

Similarity searches were performed using NCBI blast (discontiguous megablast algorithm) with four databases: “Nucleotide collection” (nr/nt), “16S ribosomal RNA sequences (Bacteria and Archaea)”, “RefSeq representative genomes”, and “High Throughput Genomic Sequences"(HTGS). Five top hits (evaluated with E-values) were listed for each database. IOLA-like 16S rRNA genes described in our previous report were excluded from the list.

Supplementary Table 4. IOLA-positive samples identified.

Case [a]	Sampling date	Age (years)	Gender	Sample type	Clinical diagnosis	Comorbidity	Steroid and immunosuppressant	Qantitative-PCR (copy/mL) [b]		Routine culture examination (BALF or EA)
								Total-16S rDNA	IOLA-16S rDNA	
KY-41 (A1)	2010.9	69	F	BALF	Pneumonia	Bronchiectasis	Prednisolone	$(2.1±0.5)×10^6$	$(1.5±0.3)×10^6$	no growth
KY-315 (A2)	2011.9	70	F	BALF	Bronchopulmonary infection	Bronchiectasis	Prednisolone	$(4.1±0.7)×10^7$	$(2.7±0.4)×10^7$	Gram-negative rod
KY-366 (A3)	2011.11	70	F	BALF	Bronchopulmonary infection	Bronchiectasis	Prednisolone	$(2.2±0.5)×10^8$	$(2.7±0.3)×10^5$	*Pseudomonas aeruginosa*
KY-405 (A4)	2011.12	71	F	BALF	Bronchopulmonary infection	Bronchiectasis	Prednisolone	$(2.1±0.5)×10^6$	$(2.5±0.3)×10^5$	*Pseudomonas aeruginosa*
KY-494 (C)	2012.6	44	M	BALF	Hospital-acquired pneumonia	Rheumatoid arthritis, Interstitial pneumonia, Diabetes mellitus	Betamethasone, Cyclosporin A	$(1.7±0.3)×10^7$	$(1.2±0.8)×10^6$	*Pseudomonas aeruginosa*
KY-90	2010.11	33	M	BALF	Community-acquired pneumonia	-	-	$(7.2±4.0)×10^6$	$(4.3±3.0)×10^3$	not analyzed
KY-207	2011.5	32	M	BALF	Healthcare-associated pneumonia	Aallergic bronchopulmonary Aspergillosis, Psychiaric disease	-	$(8.6±2.7)×10^6$	$(3.1±1.0)×10^4$	no growth
KY-282	2011.7	74	M	BALF	Healthcare-associated pneumonia	Bronchial asthma, Epilepsy	-	$(1.1±0.5)×10^8$	$(5.3±2.9)×10^4$	*Staphylococcus aureus* (MSSA) *Pseudomonas aeruginosa*
KY-440	2012.2	27	F	EA	Community-acquired pneumonia	-	-	$(2.5±0.2)×10^8$	$(3.2±2.4)×10^4$	*Haemophilus influenzae*
KY-519	2012.6	41	M	BALF	Healthcare-associated pneumonia	Bronchial asthma, Interstitial pneumonia	Prednisolone	$(6.5±1.7)×10^7$	$(5.3±1.4)×10^4$	no growth
KY-677	2013.1	72	M	EA	Healthcare-associated pneumonia	Interstitial pneumonia	Prednisolone	$(2.4±0.9)×10^8$	$(1.4±0.5)×10^5$	not analyzed
KY-798	2013.6	68	F	BALF	Non-tuberculous mycobacteria infection	Rheumatoid arthritis	Methotrexate, Salazosulfapyridine, Prednisolone	$(3.1±1.0)×10^6$	$(1.9±0.6)×10^4$	no growth
KY-923	2013.11	72	F	BALF	Bacterial bronchiolitis	Hypertension	-	$(9.0±2.9)×10^6$	$(4.3±1.3)×10^4$	*Staphylococcus aureus* (MSSA) *α- streptococcus*, *Neiseria* spp.
CL-22	2014.3	67	M	BALF	Lung abscess	-	-	$(1.5±0.2)×10^8$	$(3.5±2.0)×10^3$	not analyzed

a Labels in parentheses indicate the sample numbers used in our previous report (Fukuda et al, 2014). b The lower limits of quantification in this study were >2.4 × 10^4 copies/mL for total bacterial 16S rDNA and >2.4 × 10^2 copies/mL for IOLA 16S rDNA. In the BALF specimens from 3 patients with interstitial pneumonia (control sample from a noninfectious disease), the total 16S rDNA content was below the lower limit of quantification, and IOLA 16S rDNA was not detected. BALF; bronchoalveolar lavage fluid, EA; endotracheal aspirate.

Supplementary Table 5. Clinical information on the recurrent acute bronchopulmonary infection episodes of the patient who yielded IOLA-positive BALF specimens four times.

Episode[a]	Timing (month)	Hospitalization	Clinical symptoms record [b]	Findings of CT and/or X-ray examination	Blood tests[c]			Routine culture examination (sputum)
					CRP (mg/dL)	WBC (cells/μL)	Neutrophil (%)	
1 (KY-41)	0.0	3 weeks	Cough	Bronchiectasis, Nodular shadows	9.3	15,170	61.0	ND
2	1.7	outpatient	Purulent sputum	ND	3.1	10,140	ND	ND
3	3.5	outpatient	Purulent sputum	ND	7.0	15,380	ND	ND
4	4.9	2.5 weeks	Fever	Bronchiectasis, Nodular shadows (image not avairable)	7.0	7,460	ND	*Staphylococcus aureus* (MRSA)
5	5.3	outpatient	Purulent sputum	ND	3.8	9,610	82.6	ND
6	7.0	outpatient	Fever	ND	7.0	12,620	ND	*Pseudomonas aeruginosa*<br>*Staphylococcus aureus* (MRSA)
7	8.9	2.5 weeks	Fever	ND	7.0	12,030	ND	*Pseudomonas aeruginosa*<br>*Staphylococcus aureus* (MRSA)
8	10.0	4.5 weeks	Fever	ND	7.0	9,190	75.9	*Neiseria* spp.
9 (KY-315)	11.7	2.5 weeks	Fever, Widespread pain	Bronchiectasis, Nodular shadows	9.6	13,400	79.7	ND
10 (KY-366)	13.9	3 weeks	Fever	Bronchiectasis, Nodular shadows	18.0	12,899	ND	*Pseudomonas aeruginosa*
11 (KY-405)	15.5	3 weeks	No information	Bronchiectasis, Nodular shadows	7.2	12,710	ND	ND
12	18.1	outpatient	Purulent sputum	ND	5.5	14,080	ND	ND
13	18.7	2 weeks	No information	Bronchiectasis, Nodular shadows (image not avairable)	9.4	15,600	87.2	*Pseudomonas aeruginosa*
14	20.1	outpatient	Fever	ND	7.0	13,750	ND	ND
15	21.1	3.5 weeks	Cough, Purulent sputum	ND	7.0	9,250	73.3	*Pseudomonas aeruginosa*
16	24.0	2 weeks	Cough, Fever	Bronchiectasis,Nodular shadows (image not avairable)	7.0	19,530	95.7	*Stenotrophomonas maltophilia*<br>*Pseudomonas aeruginosa*<br>*Staphylococcus aureus* (MRSA)<br>*Candida*
17	27.4	outpatient	Fever	ND	3.6	8,020	ND	*Moraxella catarrhalis*<br>*Staphylococcus aureus* (MRSA)<br>*Neiseria* spp.
18	29.6	3 weeks	Purulent sputum	Bronchiectasis, Nodular shadows (image not avairable)	8.6	18,540	ND	*Haemophilus influenzae*
19	33.4	outpatient	No information	ND	4.3	8,750	ND	*Staphylococcus aureus* (MRSA)<br>*Neiseria* spp.
20	35.8	2 weeks	No information	Bronchiectasis, Nodular shadows (image not avairable)	11.7	14,990	ND	*Pseudomonas aeruginosa*
21	37.6	2.5 weeks	Cough, Fever, Purulent sputum	ND	12.1	16,470	ND	*Pseudomonas aeruginosa*
22	42.0	2 weeks	Fever	Bronchiectasis, Nodular shadows (image not avairable)	9.1	11,380	ND	ND

a The names of IOLA-positive BALF samples are indicated in parentheses. b “Fever” is defined as “a body temperature over 37.5°C”. c Normal ranges in this study: C-reactive protein (CRP), 0.00-0.14 mg/dL; white blood cells (WBCs), 3,300-8,600 cells/μL; and neutrophils, 42.4-75.0%. ND, not done. MRSA, methicillin-resistant Staphylococcus aureus.

Supplementary Table 6. List of the accession numbers of the 121 PPases in this study.

AAF01029.1_Pyrobaculum_aerophilum	YP_181516.1Dehalococcoides_mccartyi_195	YP_921602.1_Nocardioides_sp._JS614
YP_001037849.1_Ruminiclostridium_thermocellum_ATCC_27405	ZP_03906694.1_Denitrovibrio_acetiphilus_DSM_12809	ZP_03727379.1_Diplosphaera_colitermitum_TAV2
ZP_04453789.1_Abiotrophia_defectiva_ATCC_49176	YP_521069.1_Desulfitobacterium_hafniense_Y51	NP_001054701.1_Oryza_sativa
BAA83103.1_Acetabularia_acetabulum	YP_001530700.1_Desulfococcus_oleovorans_Hxd3	CAL51797.1_Ostreococcus_tauri
ZP_04874733.1_Aciduliprofundum_boonei_T469	ZP_01313190.1_Desulfuromonas_acetoxidans_DSM_684	CAL50249.1_Ostreococcus_tauri
YP_001878130.1_Akkermansia_muciniphila_ATCC_BAA-835	ZP_03729322.1_Dethiobacter_alkaliphilus_AHT_1	ZP_04577892.1_Oxalobacter_formigenes_HOxBLS
ZP_05799147.1_Anaerobaculum_hydrogeniformans_ATCC_BAA-1850	YP_002251142.1_Dictyoglomus_thermophilum_H-6-12	ZP_02094589.1_Parvimonas_micra_ATCC_33270
ZP_02861667.1_Anaerofustis_stercorihominis_DSM_17244	ZP_01995168.1Dorea_longicatena_DSM_13814	YP_001213266.1_Pelotomaculum_thermopropionicum_SI
ZP_02417489.1_Anaerostipes_caccae_DSM_14662	YP_003086371.1_Dyadobacter_fermentans_DSM_18053	EER08598.1_Perkinsus_marinus_ATCC_50983
AAA32754.1_Arabidopsis_thaliana	YP_457403.1_Erythrobacter_litoralis_HTCC2594	XP_002178316.1_Phaeodactylum_tricornutum
AAC83018.1_Arabidopsis_thaliana	YP_001410846.1_Fervidobacterium_nodosum_Rt17-B1	XP_002183861.1_Phaeodactylum_tricornutum_
ZP_04771156.1_Asticcacaulis_excentricus_CB_48	ZP_03700932.1_Flavobacteria_bacterium_MS024-3C	ZP_01852129.1_Planctomyces_maris_DSM_8797
ZP_03463001.1_Bacteroides_pectinophilus_ATCC_43243	YP_001193830.1_Flavobacterium_johnsoniae_UW101	AAG21366.1_Plasmodium_falciparum
YP_001299560.1_Bacteroides_vulgatus_ATCC_8482	ZP_04831980.1_Gallionella_ferruginea_ES-2	AAD17215.1_Plasmodium_falciparum
NP_968591.1_Bdellovibrio_bacteriovorus_HD100	NP_954331.1_Geobacter_sulfurreducens_PCA	ZP_01910339.1_Plesiocystis_pacifica_SIR-1
ZP_03783250.1_Blautia_hydrogenotrophica_DSM_10507	ZP_03892174.1_Geodermatophilus_obscurus_DSM_43160	ZP_01118223.1_Polaribacter_irgensii_23-P
YP_002721534.1_Brachyspira_hyodysenteriae_WA1	ZP_03880259.1_Haliangium_ochraceum_DSM_14365	ZP_05734912.2_Prevotella_tannerae_ATCC_51259
NP_771666.1_Bradyrhizobium_diazoefficiens_USDA_110	YP_002509320.1_Halothermothrix_orenii_H_168	AAC38615.2_Rhodospirillum_rubrum_ATCC_11170
YP_001540035.1_Caldivirga_maquilingensis_IC-167	YP_001679270.1_Heliobacterium_modesticaldum_Ice1	ZP_04423801.1_Rhodothermus_marinus_DSM_4252
YP_001540771.1_Caldivirga_maquilingensis_IC-167	YP_759877.1_Hyphomonas_neptunium_ATCC_15444	YP_003196855.1_Robiginitalea_biformata_HTCC2501
YP_001957756.1_Candidatus_Amoebophilus_asiaticus_5a2	ZP_05861386.1_Jonquetella_anthropi	YP_444948.1_Salinibacter_ruber_DSM_13855
YP_903362.1_Candidatus_Ruthia_magnifica	YP_002939864.1Kosmotoga_olearia_TBF_19.5.1	ZP_04455192.1_Shuttleworthia_satelles_DSM_14600
ZP_03392450.1_Capnocytophaga_sputigena	YP_594877.1_Lawsonia_intracellularis	YP_001616292.1_Sorangium_cellulosum_So_ce56
YP_359158.1_Carboxydothermus_hydrogenoformans_Z-2901	YP_001840784.1_Leptospira_biflexa_	ZP_04490407.1_Spirosoma_linguale_DSM_74
YP_003119112.1_Catenulispora_acidiphila_DSM_44928	NP_711652.2_Leptospira_interrogans_serovar_Lai_str._56601	ZP_04482946.1_Stackebrandtia_nassauensis_DSM_44728
NP_420176.1_Caulobacter_vibrioides_CB15	NP_108517.1_Mesorhizobium_loti_MAFF303099	NP_627745.1_Streptomyces_coelicolor_A3(2)
ZP_04367522.1_Cellulomonas_flavigena_DSM_20109	YP_565684.1_Methanococcoides_burtonii_DSM_6242	YP_076391.1_Symbiobacterium_thermophilum_IAM_14863
YP_875904.1_Cenarchaeum_symbiosum_A	NP_632724.1_Methanosarcina_mazei_Go1	YP_461937.1_Syntrophus_aciditrophicus_SB
YP_003125805.1_Chitinophaga_pinensis_DSM_2588	NP_632725.1_Methanosarcina_mazei_Go1	YP_003074236.1_Teredinibacter_turnerae_T7901
CAC44451.1_Chlamydomonas_reinhardtii	YP_503835.1_Methanospirillum_hungatei_JF-1	XP_001020551.1_Tetrahymena_thermophila_SB210
YP_001943220.1_Chlorobium_limicola_DSM_245	YP_001940861.1_Methylacidiphilum_infernorum_V4	ZP_03856559.1_Thermobaculum_terrenum_ATCC_BAA-798
YP_001634923.1_Chloroflexus_aurantiacus	YP_001021312.1_Methylibium_petroleiphilum_PM1	YP_002521938.1_Thermomicrobium_roseum_DSM_5159
ZP_03127672.1__Chthoniobacter_flavus	YP_001640547.1_Methylobacterium_extorquens_PA1	YP_001471635.1_Thermotoga_lettingae_
ZP_02088591.1_Clostridium_bolteae_ATCC_BAA-613	AAU92464.1_Methylococcus_capsulatus_	YP_001470939.1_Thermotoga_lettingae_TM
YP_001921624.1_Clostridium_botulinum	ZP_05105141.1_Methylophaga_thiooxidans_DMS010	YP_001471565.1_Thermotoga_lettingae_TMO
ZP_02078667.1_Clostridium_leptum_DSM_753	ZP_01551779.1_Methylophilales_bacterium_HTCC2181	NP_227989.1_Thermotoga_maritima_MSB8
NP_781083.1_Clostridium_tetani_E88	EEH58547.1_Micromonas_pusilla_CCMP1545	AAK38076.1_Toxoplasma_gondii
YP_002246899.1_Coprothermobacter_proteolyticus_DSM_5265	YP_430205.1_Moorella_thermoacetica_ATCC_39073	XP_814868.1_Trypanosoma_cruzi_strain_CL_Brener
BAE19660.1_Cyanidioschyzon_merolae	YP_429292.1_Moorella_thermoacetica_ATCC_39073	BAA23649.1_Vigna_radiata
YP_286403.1_Dechloromonas_aromatica_RCB	ZP_01127763.1_Nitrococcus_mobilis_Nb-231	
YP_181498.1Dehalococcoides_mccartyi_195	NP_841957.1_Nitrosomonas_europaea_ATCC_19718	
