# Supplementary material for: A human respiratory tract-associated bacterium with an extremely small genome
Source: Commun Biol. 2021 May 26;4:628. doi: 10.1038/s42003-021-02162-6 (PMC8155191; doi:10.1038/s42003-021-02162-6)
Supplement: Supplementary file 3 — Description of Additional Supplementary Files [file 42003_2021_2162_MOESM3_ESM.pdf]

Description of Additional Supplementary Files

File name: Supplementary Data 1

Description: List of the CDSs identified in the IOLA genome.

File name: Supplementary Data 2

Description: Information on the genomes of all microorganisms used in the phylogenetic analyses. The datasets of housekeeping protein sequences and ribosomal RNA gene sequences used in the phylogenetic analyses were collected from the Joint Genome Institute's IMG/M database (https://img.jgi.doe.gov). The classification information (phylum, class, order, family, genus and species), IMG genome ID, and sequencing status are listed according to each analysis.

File name: Supplementary Data 3

Description: A list of host-associated bacteria with small genomes (under 1.2 Mb) used for the comparison analysis with IOLA. Human-associated bacteria are colored orange. The clusters were assigned based on the heatmap of COG functional category proportions in Fig. 5.

File name: Supplementary Data 4

Description: Comparison of gene content between IOLA and 39 host-associated bacteria with small genomes and low GC contents.

File name: Supplementary Data 5

Description: Primer sequences used for the amplification and sequencing of 12 genome segments of IOLA.
