# Supplementary material for: A human respiratory tract-associated bacterium with an extremely small genome
Source: Commun Biol. 2021 May 26;4:628. doi: 10.1038/s42003-021-02162-6 (PMC8155191; doi:10.1038/s42003-021-02162-6)
Supplement: Supplementary file 9 — Reporting Summary [file 42003_2021_2162_MOESM9_ESM.pdf]

nature research

Corresponding author(s): Kazumasa Fukuda and Tetsuya Hayashi
COMMSBIO-20-2931B

Last updated by author(s): Apr 3, 2021

Reporting Summary

Nature Research wishes to improve the reproducibility of the work that we publish. This form provides structure for consistency and transparency in reporting. For further information on Nature Research policies, see our Editorial Policies and the Editorial Policy Checklist.

Statistics

For all statistical analyses, confirm that the following items are present in the figure legend, table legend, main text, or Methods section.

n/a	Confirmed	
☒	☐	The exact sample size (*n*) for each experimental group/condition, given as a discrete number and unit of measurement
☒	☐	A statement on whether measurements were taken from distinct samples or whether the same sample was measured repeatedly
☒	☐	The statistical test(s) used AND whether they are one- or two-sided<br>*Only common tests should be described solely by name; describe more complex techniques in the Methods section.*
☒	☐	A description of all covariates tested
☒	☐	A description of any assumptions or corrections, such as tests of normality and adjustment for multiple comparisons
☐	☒	A full description of the statistical parameters including central tendency (e.g. means) or other basic estimates (e.g. regression coefficient) AND variation (e.g. standard deviation) or associated estimates of uncertainty (e.g. confidence intervals)
☒	☐	For null hypothesis testing, the test statistic (e.g. *F*, *t*, *r*) with confidence intervals, effect sizes, degrees of freedom and *P* value noted<br>*Give P values as exact values whenever suitable.*
☒	☐	For Bayesian analysis, information on the choice of priors and Markov chain Monte Carlo settings
☒	☐	For hierarchical and complex designs, identification of the appropriate level for tests and full reporting of outcomes
☒	☐	Estimates of effect sizes (e.g. Cohen's *d*, Pearson's *r*), indicating how they were calculated

Our web collection on statistics for biologists contains articles on many of the points above.

Software and code

Policy information about availability of computer code

Data collection	No software was used for data collection in this study.
Data analysis	FastQC, SPAdes, Platanus, and ATSQ5.1.3 were used for a genome sequence assemble. MiGAP(http://www.migap.org/),MetaGeneAnnotator ver. 1.0, and tRNAscan-SE ver. 1.23 were used to identify CDSs and tRNA genes. NCBI blastp, CD-search, Pfam, and TIGRfam were used for annotation of CDSs. SOSUI and PSORTb were used to predict secondary structures and subcellular localization of the CDSs. R software ver. 3.5.0 (http://cran.r-project.org) was used for a clustering analysis. MAFFT,Gblocks 0.91b, BMGE version 1.12, IQ-tree, MrBayes 3.2.6, MEGA 7.0.26, and iTOL v 5.5.1 were used for phylogenetic analyses.

For manuscripts utilizing custom algorithms or software that are central to the research but not yet described in published literature, software must be made available to editors and reviewers. We strongly encourage code deposition in a community repository (e.g. GitHub). See the Nature Research guidelines for submitting code & software for further information.

Data

Policy information about availability of data

All manuscripts must include a data availability statement. This statement should provide the following information, where applicable:

- Accession codes, unique identifiers, or web links for publicly available datasets
- A list of figures that have associated raw data
- A description of any restrictions on data availability

The sequence read data of IOLA KY-405 have been deposited in DDBJ Sequence Read Archive (DRA) under the BioProject identifier PRJDB7462. The assembled IOLA genome sequence has been deposited in DDBJ/EMBL/GenBank under accession No. AP019313. The 16S rRNA gene sequences (accession No. LC435447-LC435456) and PCR amplicon sequences (accession No. LC438941-LC439108) from the IOLA-positive specimens have also been deposited in DDBJ/EMBL/GenBank.

Field-specific reporting

Please select the one below that is the best fit for your research. If you are not sure, read the appropriate sections before making your selection.

- [x] Life sciences
- [ ] Behavioural & social sciences
- [ ] Ecological, evolutionary & environmental sciences

For a reference copy of the document with all sections, see nature.com/documents/nr-reporting-summary-flat.pdf

Life sciences study design

All studies must disclose on these points even when the disclosure is negative.

Sample size	The 523 specimens comprised 479 BALF and 44 sputum samples obtained from the University of Occupational and Environmental Health (UOEH), Japan Hospital, and its referring hospitals between September 2010 and August 2016 were used in this study.
Data exclusions	No data was excluded.
Replication	This is not applicable to this study.
Randomization	This is not applicable to this study.
Blinding	This is not applicable to this study.

Reporting for specific materials, systems and methods

We require information from authors about some types of materials, experimental systems and methods used in many studies. Here, indicate whether each material, system or method listed is relevant to your study. If you are not sure if a list item applies to your research, read the appropriate section before selecting a response.

Materials & experimental systems

n/a	Involved in the study
x	Antibodies
x	Eukaryotic cell lines
x	Palaeontology and archaeology
x	Animals and other organisms
	x Human research participants
x	Clinical data
x	Dual use research of concern

Methods

n/a	Involved in the study
x	ChIP-seq
x	Flow cytometry
x	MRI-based neuroimaging

Human research participants

Policy information about studies involving human research participants

Population characteristics	A total of 523 clinical specimens comprised 479 BALF and 44 sputum samples were obtained from the University of Occupational and Environmental Health (UOEH), Japan Hospital, and its referring hospitals between September 2010 and August 2016.
Recruitment	The patients who were performed bronchoscopy to evaluate the causative pathogens in the lesions, and provided written informed consent, were enrolled in this study.
Ethics oversight	This study was approved by the human and animal ethics review committee of UOEH (No. 09-118).

Note that full information on the approval of the study protocol must also be provided in the manuscript.
